# UCP-3 uncoupling protein confers hypoxia resistance to renal epithelial cells and is upregulated in renal cell carcinoma

**DOI:** 10.1038/srep13450

**Published:** 2015-08-25

**Authors:** Norbert Braun, Dominik Klumpp, Jörg Hennenlotter, Jens Bedke, Christophe Duranton, Martin Bleif, Stephan M. Huber

**Affiliations:** 1 Department of Radiation Oncology, University of Tübingen Germany.; 2 Department of Urology, University of Tübingen, Germany; 3 Department of Faculté de Médecine, Université de Nice-Sophia Antipolis, Nice, France

## Abstract

Tumor cells can adapt to a hostile environment with reduced oxygen supply. The present study aimed to identify mechanisms that confer hypoxia resistance. Partially hypoxia/reoxygenation (H/R)-resistant proximal tubular (PT) cells were selected by exposing PT cultures to repetitive cycles of H/R. Thereafter, H/R-induced changes in mRNA and protein expression, inner mitochondrial membrane potential (ΔΨ_m_), formation of superoxide, and cell death were compared between H/R-adapted and control PT cultures. As a result, H/R-adapted PT cells exhibited lower H/R-induced hyperpolarization of ΔΨ_m_ and produced less superoxide than the control cultures. Consequently, H/R triggered ΔΨ_m_ break-down and DNA degradation in a lower percentage of H/R-adapted than control PT cells. Moreover, H/R induced upregulation of mitochondrial uncoupling protein-3 (UCP-3) in H/R-adapted PT but not in control cultures. In addition, ionizing radiation killed a lower percentage of H/R-adapted as compared to control cells suggestive of an H/R-radiation cross-resistance developed by the selection procedure. Knockdown of UCP-3 decreased H/R- and radioresitance of the H/R-adapted cells. Finally, UCP-3 protein abundance of PT-derived clear cell renal cell carcinoma and normal renal tissue was compared in human specimens indicating upregulation of UCP-3 during tumor development. Combined, our data suggest functional significance of UCP-3 for H/R resistance.

Intermittent or chronic hypoxia due to insufficient vascularization and tissue malperfusion is a common feature of human solid tumors. In anti-cancer therapy, tumor hypoxia is a severe risk factor since the causative malperfusion limits not only oxygen supply but also the perfusion of the tumor with chemotherapeutics. In addition, low oxygen pressure during radiation therapy decreases the number of ionizing radiation-induced DNA double strand breaks and thereby the therapy efficacy by a factor of up to 3. Moreover, adaptation of tumor cells to a malperfused hypoxic microenvironment often induces/selects tumor cells with higher malignancy, metastatic potential and intrinsic resistance to radiation or chemotherapy. For instance, upregulation of highly efficient Na^+^-coupled glucose uptake by several tumor entities does not only ensure glucose supply by the malperfused glucose-depleted environment but also confer radioresistance^1^,^2^,^3^.

Hypoxia and reoxygenation may result in oxidative insults as reported for ischemia/reperfusion injury of the heart. During reoxygenation of hypoxic tissue, oxidative stress may be triggered by Ca^2+^ overload of the mitochondria, concomitant hyperpolarization of the voltage (ΔΨ_m_) across the inner mitochondrial membrane and superoxide formation as a consequence thereof (for review see Ref. 4). Tumor cells surrounded by a microenvironment with varying oxygen pressure are, therefore, under continuous risk of mitochondria-derived oxidative insults.

The present study aimed to define mechanisms of hypoxia/reoxygenation (H/R) adaptation *in vitro* by comparing H/R-adapted with highly hypoxia-sensitive parental cells. For H/R adaptation, immortalized primary cultures of mouse proximal convoluted tubule (PT) which are highly dependent on oxidative respiration and, therefore, highly hypoxia-sensitive were subjected to repeated cycles of hypoxia and reoxygenation^5^. That way H/R-adapted PT cultures were then compared with the continuously normoxic-grown parental control cells in terms of H/R-induced impairment of mitochondrial function, formation of reactive oxygen species (ROS), cell death and gene expression. Our data suggest that up-regulation of the mitochondrial uncoupling protein-3 (UCP-3) contributes to H/R adaptation *in vitro*. Finally, to estimate whether this *in vitro* finding might be translated to the *in vivo* situation, the present study analyzed UCP-3 expression in PT-derived clear cell renal cell carcinoma demonstrating marked up-regulation of UCP-3 by the tumor cells.

## Results

### Selection of partial H/R-resistant proximal convoluted tubule (PT) cells

**Figure 1:**
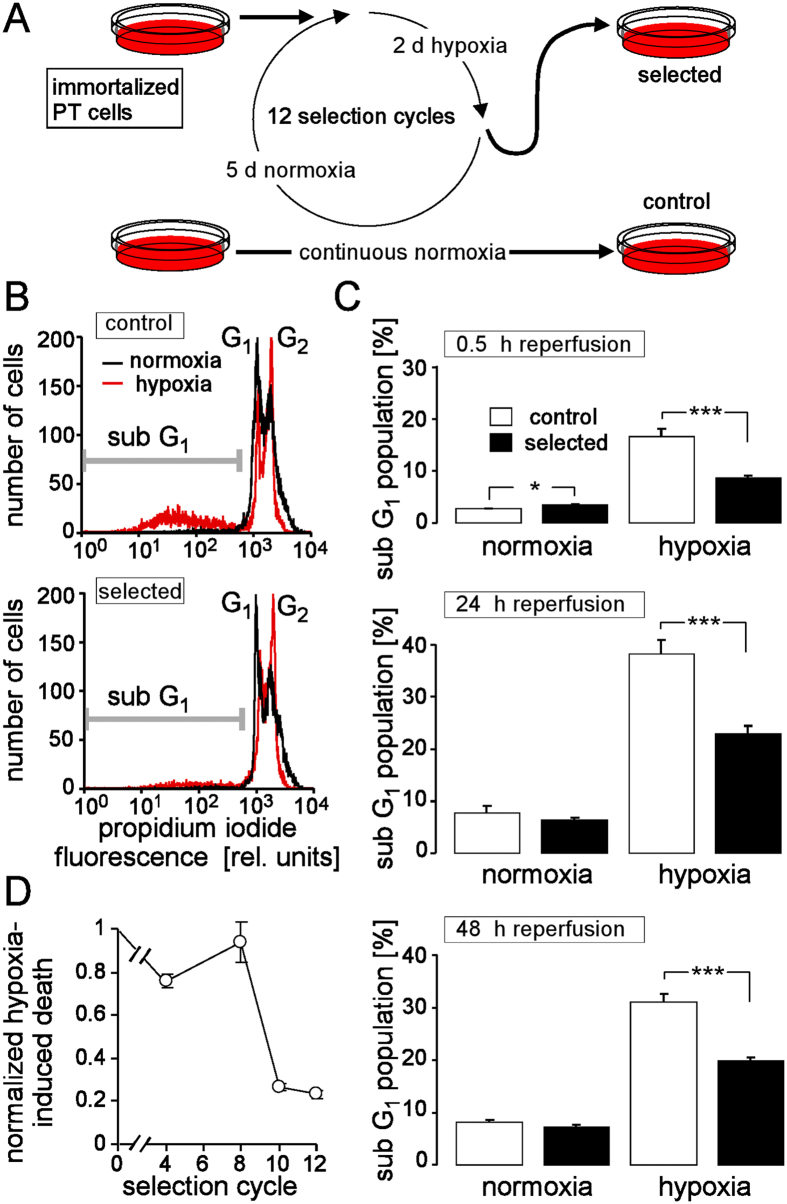
Repetitive exposure to hypoxia/reoxygenation (H/R) selected partial H/R-resistent proximal convoluted tubule (PT) cells. (**A**) Selection protocol. (**B**) Histograms showing the propidium iodide fluorescence intensity of permeabilized control and H/R-adapted PT cells. Cells were recorded by flow cytometry either under control conditions (72 h of normoxia, black line) or after 48 h of hypoxia (0.1% oxygen) followed by 24 h of reoxygenation (red line). The marker indicates the dead cells (sub G_1_ population). (**C**) Mean percentage (±SE, n = 24 from 4 cultures each measured in hexaduplicate) of dead cells (sub G_1_ population) in control (open bars) and H/R-adapted cultures (closed bars) grown under normoxia (left) or under hypoxia (48 h of 0.1% oxygen) followed by 0.5 h (top), 24 h (middle) and 48 h (bottom) of reoxygenation. * and *** indicate p ≤ 0.05 and p ≤ 0.001, respectively (ANOVA). (**D**) Time course of resistance acquisition. Shown is the selection-cycle-dependent H/R-induced cell death of the H/R-adapted cultures normalized to that of the particular control cultures (means ± SE, n = 4).

Four parallel cultures of PT cells were passaged (once per week) for 12 weeks. During this period of time, cells where subjected to weekly cycles of hypoxia (0.1% oxygen for 48 h) and reoxygenation (5 days). Each cycle started 2–3 d after passaging the cells. As a control, further four PT cultures were grown under continuous normoxia, and passaged twice weekly for 12 weeks (Fig. 1A). Thereafter, all cultures were passaged twice to increase the cell number, aliquoted and frozen. To test for an acquired H/R resistance, sub-confluent H/R-adapted and control cultures were grown for 48 h under normoxia or hypoxia (0.1% oxygen) followed by 0.5, 24 or 48 h of reoxygenation. Thereafter, the DNA of the cells was stained with propidium iodide (Nicoletti protocol). As shown in the histograms of Fig. 1B, H/R induced a G_2_/M cell cycle arrest in both, control and H/R-adapted PT cells, suggestive of H/R-caused genotoxic stress. In addition, H/R resulted in cell death as defined by the sub G_1_ population of the propidium iodide histogram. Cell death induction was dependent on reoxygenation time (Fig. 1B). Most importantly, cell death was significantly reduced in the H/R-adapted cells as compared to the control cultures (Fig. 1C) indicating acquisition of a partial H/R resistance during the selection time. Fig. 1D shows the selection cycle-dependent acquisition of the partial H/R resistance.

### H/R-induced hyperpolarization of the inner mitochondrial membrane potential (ΔΨ_m_)

**Figure 2:**
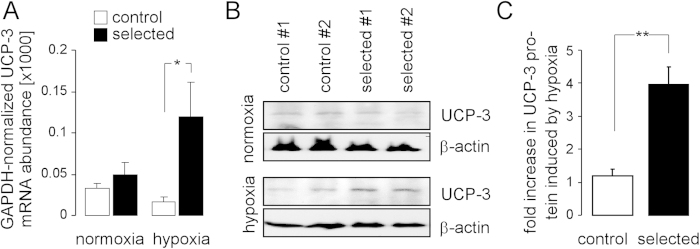
H/R induces an up-regulation of the mitochondrial uncoupling protein-3 (UCP-3) in H/R-adapted but not in control PT cultures. (**A**) Mean (±SE, n = 3) GAPDH-normalized UCP-3 mRNA abundance of control (open bars) and H/R-adapted PT cultures (closed bars) under normoxia (left) or after hypoxia (48 h)/reoxygenation (24 h) as determined by quantitative RT-PCR. (**B**) Immunoblot of PAGE-separated proteins from control (1^st^ and 2^nd^ lane) H/R-adapted PT cultures (3^rd^ and 4^th^ lane) probed against UCP-3 and β-actin. Cell lysates were prepared from normoxic (upper blot) and cultures which underwent H/R stress (lower blot). (**C**). Densitometrically semi-quantified increase in UCP-3 protein of control (open bar) and H/R-adapted PT-cultures induced by hypoxia(48 h)/reoxygenation (24 h; means ± SE, n = 3 cultures each; * indicates p ≤ 0.05, two-tailed Welch-corrected t-test).

Reportedly, reoxygenation may be associated with hyperpolarization of ΔΨ_m_. We, therefore, tested for the effect of hypoxia (48 h)/reoxygenation (24 h) on ΔΨ_m_ in control and H/R-adapted PT-cultures by flow cytometry applying the voltage-sensitive fluorescence dye TMRE. H/R induced break-down of ΔΨ_m_ (Fig. 2) in a significant cell fraction confirming H/R-induced cell death (Fig. 2A–C). In the surviving cells, on the other hand, H/R hyperpolarized ΔΨ_m_ (Fig. 2B,D). Markedly, H/R-adapted PT cultures exhibited both, significantly less H/R-induced ΔΨ_m_ break-down (Fig. 2C) and significantly less ΔΨ_m_ hyperpolarization in the surviving cell population (Fig. 2D).

### H/R-induced formation of reactive oxygen species (ROS)

**Figure 3:**
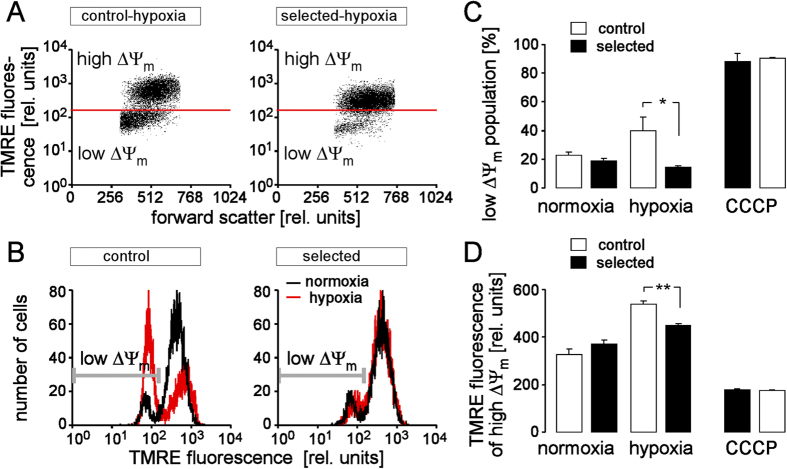
H/R-adapted cultures exhibit after H/R stress less hyperpolarization of the inner mitochondrial membrane potential (ΔΨ_m_) than control cultures. (**A,B**) Dot plots (**A**) and histograms (**B**) showing forward scatter and tetramethylrhodamine-ethyl-ester-perchlorate (TMRE) fluorescence as a measure of cell size and ΔΨ_m_, respectively. Depicted are a control (left) and a H/R-adapted PT culture (right) recorded by flow cytometry under normoxic conditions (black lines in (**B**)) and after H/R stress (48 h hypoxia/24 h reoxygenation; (**A**) and red histograms in (**B**)). Cell populations with dissipated ΔΨ_m_ (low ΔΨ_m_) are indicated by gate and marker in A and B, respectively. (**C,D**) Mean percentage of control (open bars) and H/R-adapted cells (closed bars) with broken-down ΔΨm (**C**) and (**D**) mean TMRE fluorescence intensity of the cell population with high ΔΨ_m_ (±SE, n = 9 from 3 cultures each determined in triplicate) recorded as in (**B**) under normoxic conditions (left), after H/R stress (48 h hypoxia/24 h reoxygenation, middle), or after pharmacological break-down of ΔΨ_m_ by the proton ionophore carbonyl cyanide-3-chlorophenylhydrazone (CCCP, 1 μM). * and ** indicate p ≤ 0.05 and p ≤ 0.01, respectively (ANOVA).

Mitochondrial superoxide formation increases with hyperpolarization of ΔΨ_m_. Next, we analyzed cumulative superoxide formation of the control and selected cultures during normoxia and upon hypoxia (48 h)/reoxygenation (24 h) by flow cytometry using the redox-sensitive fluorescence dye MitoSOX. As shown in Fig. 3, H/R-induced superoxide formation was significantly lower in H/R-adapted than in control PT cultures.

### Function of Uncoupling Protein-3 (UCP-3)

**Figure 4:**
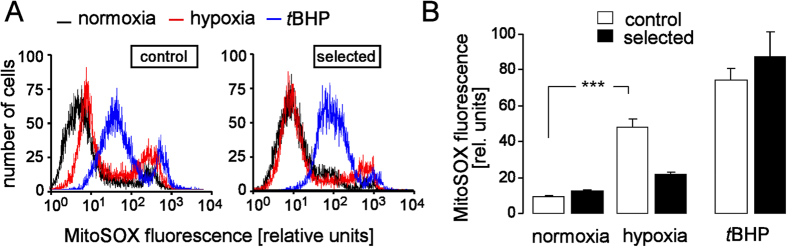
H/R produces less reactive oxygen species (ROS) in H/R-adapted than in control cultures. (**A**) Histograms showing the MitoSOX fluorescence as a measure of mitochondrial superoxide production. PT cells were recorded by flow cytometry from a control (top) and a H/R-adapted culture under normoxic conditions (black lines), after H/R stress (48 h hypoxia/24 h reoxygenation, red lines) or after oxidation with *tert*butylhydroperoxide (*t*BHP, 1 mM). (**B**) Mean MitoSOX fluorescence intensities recorded as in (**A**) under normoxic conditions (left), after H/R stress (48 h hypoxia/24 h reoxygenation, middle, data are means ± SE, n = 10–12 from 4 cultures each determined in duplicate or triplicate), or after oxidation with *t*BHP (means ± SE, n = 4 from 4 cultures recorded under normoxia) *** indicates p ≤ 0.001 (ANOVA).

To define candidate genes that might confer H/R resistance, mRNA abundances (quantitative RT-PCR microarrays) were compared between control and H/R-adapted PT cultures after normoxic culture conditions and upon hypoxia (48 h)/reoxygenation (24 h). Several gene transcripts involved in apoptosis and antioxidative defense seemingly differed in abundance between the control and H/R-adapted PT cultures (see Supplementary Material, Fig. I) suggesting that long-term H/R adaptation was accompanied by upregulation of oxidative defense, DNA-repair and the apoptosis inhibitor survivin on the one hand, and by an enhancement of the apoptotic cell death machinery on the other. In addition, H/R-adapted PT cultures upregulated uncoupling protein-3 (UCP-3) mRNA, in particular, upon acute H/R (Fig 4A). Western blotting experiments confirmed that H/R induced a significant upregulation of UCP-3 protein expression (~4-fold increase) in the H/R-adapted but not in the control cultures (Fig. 4B,C).

**Figure 5:**
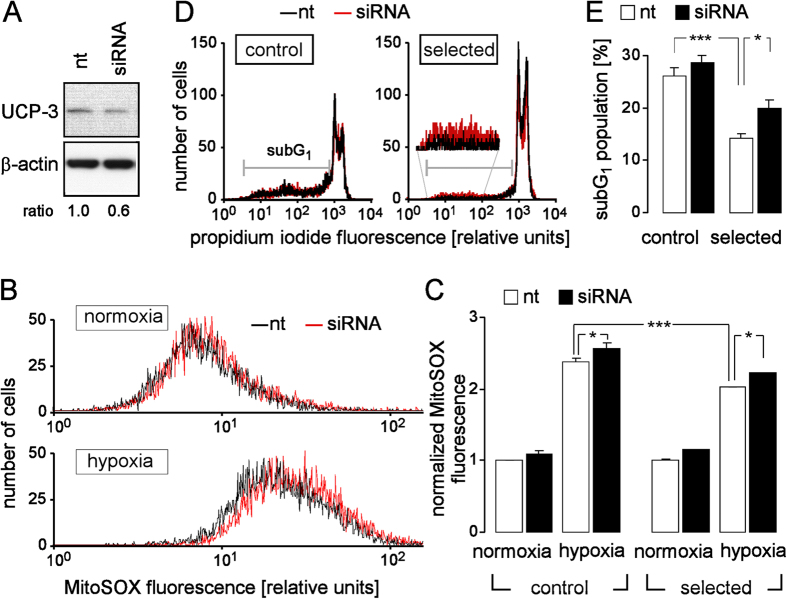
UCP-3 knock-down increases H/R-induced superoxide production and cell death. (**A**) Immunoblot showing the UCP-3 (upper gel) and—for loading control—the β-actin (lower gel) protein abundance in PT culture transfected with non-targeting (nt) RNA (left lane) or with UCP-3 siRNA (right lane). The ratio indicates the densitometrically semi-quantified β-actin-normalized relative UCP-3 protein abundance. (**B**) Histograms showing the MitoSOX fluorescence of nt- (black lines) and UCP-3 siRNA-transfected (red lines) PT cells after normoxia (top) or H/R stress (48 h hypoxia/24 h reoxygenation, bottom), recorded as in Fig. 4. (C) Mean normalized MitoSOX fluorescence intensities of nt- (open bars) and UCP-3 siRNA-transfected (closed bars) control and H/R-adapted PT cultures PT cells recorded in as in (**B**) under normoxic conditions or after H/R stress. Data are means ± SE, n = 6 from 2 cultures each determined in triplicate. (**D**) Histograms showing the propidium iodide fluorescence of permeabilized nt- (black line) and UCP-3 siRNA-transfected (red line) control (left) and H/R-adapted PT cultures (right) after hypoxia (48 h)/reoxygenation (24 h, recorded as in Fig. 1). (**E**) Mean percentage (±SE, n = 7–9 from 3 cultures each recorded in duplicate or triplicate) of dead cells (sub G_1_ population) in nt- (open bars) or UCP-3 siRNA-transfected control and H/R-adapted PT cultures after H/R stress (48 h hypoxia/24 h reoxygenation. * and *** indicate p ≤ 0.05 and p ≤ 0.001, respectively (ANOVA).

Since UCP-3 has been demonstrated to reduce mitochondrial ROS formation, to lower ischemia/reperfusion insults, and to become up-regulated by anaerobic muscle exercise (see discussion), upregulation of UCP-3 might directly contribute to the observed partial H/R resistance. Therefore, we knocked-down UCP-3 in control and H/R-adapted cultures by RNA interference (Fig 5A) and determined mitochondrial superoxide formation and the the sub G_1_ fraction of propidium iodide-stained cells after normoxia or hypoxia (48 h)/reoxygenation (24 h). As shown in Fig. 5, a knock-down-mediated decrease by ~40% of UCP-3 protein abundance (Fig. 5A) significantly increased mitochondrial superoxide formation (Fig. 5B,C) in control and H/R-adapted PT cultures. Moreover UCP-3 knock-down lowered survival after H/R of the H/R-adapted but not of the control PT cultures (Fig. 5D,E). This suggests that UCP-3 upregulation contributes to the H/R adaptation.

### UCP expression in renal cell carcinoma

**Figure 6:**
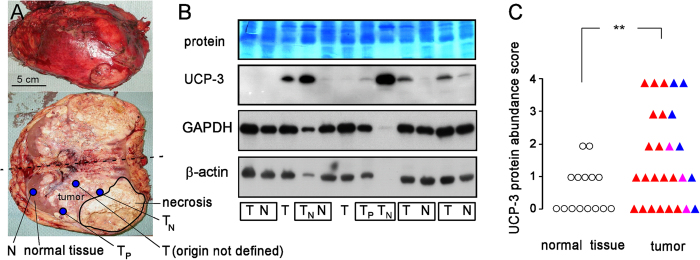
UCP-3 protein expression in human renal cell carcinoma and adjacent non-cancerous normal renal tissue. (**A**) Human nephrectomy specimen top view (upper panel) and opened cut halves (lower panel) with renal cell carcinoma (RCC). Areas of necrosis and residual normal tissue, as well as locations of the sample taking are indicated (N: normal renal tissue, T: tumor without defined origin. T_N_: tumor close to necrotic area, T_P_: tumor at the periphery). (**B**) Immunoblot of renal cell carcinoma and normal renal tissue (as shown in (**A**)) probed against UCP-3 (2^nd^ panel), GAPDH (3^rd^ panel) and β-actin (4^th^ panel). The 1^st^ panel depicts the corresponding protein stain (PageBlue). The low or even missing housekeeper bands of the 4^th^ and 8^th^ sample (from left) might be explained by strong overexpression of other proteins by theses two tumors. Since electrophoresis loading volume was adjusted to total protein concentration strong overexpression of some proteins dilutes the housekeeper proteins in these samples. Boxes indicate samples which originated from the same kidney. (**C**) Scoring of the UCP-3 protein abundance in non-cancerous renal tissue (open circles) and clear cell RCCs (closed red triangles), papillary RCCs (closed blue triangles) or mixed RCCs (closed violet triangles). ** indicates p ≤ 0.01, Welch-corrected, two-tailed t-test.

To test whether UCP-3 might fulfill similar function *in vivo*, UCP-3 protein abundance was determined by immunoblotting in frozen specimens from human renal cell carcinoma (RCC) and normal renal tissue. Twentyseven tumor specimens (18 clear cell RCCs, ccRCS, 6 papillary RCCs, pRCCs, and 3 mixed clear cell/papillary RCCs, median histological grading G = 2) as well as 15 non-cancerous renal specimens were selected form 21 patients that underwent (partial) nephrectomy. The patients (11 men, 10 women, mean age = 67 ± 2 years) developed large tumors ranging from 3 cm to 21 cm in diameter (mean diameter = 8 ± 1 cm). These tumors had large necrotic areas pointing to insufficient vascularization (Fig. 6A). The majority of tumor specimens showed various levels of UCP-3 protein abundance (Fig. 6B) that was on average significantly higher than in the non-cancerous renal samples (Fig. 6C) indicating upregulation of UCP-3 during tumor development. Although some tumor specimens close to necrotic tumor areas (i.e., from presumed hypoxic regions, T_N_ in Fig. 6B) showed extremely high UCP-3 protein abundance while peripheral tumor (T_P_ in Fig. 6B) exhibited very low expression, the average UCP-3 abundance of T_N_ specimens was not significantly different from that of the tumor samples of non-defined origin (data not shown). No difference in UCP-3 protein abundance was found between ccRCCs (Fig. 6C, red triangles) and pRCCs (Fig. 6C, blue triangles). In addition, univariate testing of the data suggested that UCP-3 abundance was not associated with patients’ age, gender, tumor size, occurrence of lymph node or distant metastasis, or histological tumor grading (data not shown).

To test whether RCCs similarly to UCP-3 upregulate other mitochondrial uncoupling proteins, UCP-1 and UCP-2 protein abundance was determined by immunoblotting in the RCC resection material (Suppl Fig. IIA). The results suggested downregulation of UCP-1 by all and upregulation of UCP-2 by some of the tested RCCs. In addition, the ccRCC database of The Cancer Genome Atlas (TCGA) was queried for UCP-1, -2, and 3 mRNA expression of the tumor and survival of the ccRCC patients (Suppl. Fig. IIB, C). The TCGA data suggest co-occurrence of high abundant UCP-2 and UCP3 mRNA in the ccRCC specimens (data not shown) as well as shorter survival of ccRCC patients with high UCP-2 or UCP-3 mRNA abundance in the tumors as compared to the patients with “middle-rate” UCP expressions (Suppl. Fig. IIB, C). High abundance of UCP-1 mRNA, by contrast, was not associated with altered survival of the ccRCC patients. Since subgroup analysis concerning tumor staging, treatment regimes, etc. could not be performed, the conclusions drawn from the TCGA data is constrained. Nevertheless, the observed associations might point to a prognostic value of the UCP-3 or UCP-2 expression by the ccRCC. In addition, the data might hint to a functional redundancy of UCP-2 and UCP-3.

### H/R-adapted cultures exhibit higher resistance against ionizing radiation

**Figure 7:**
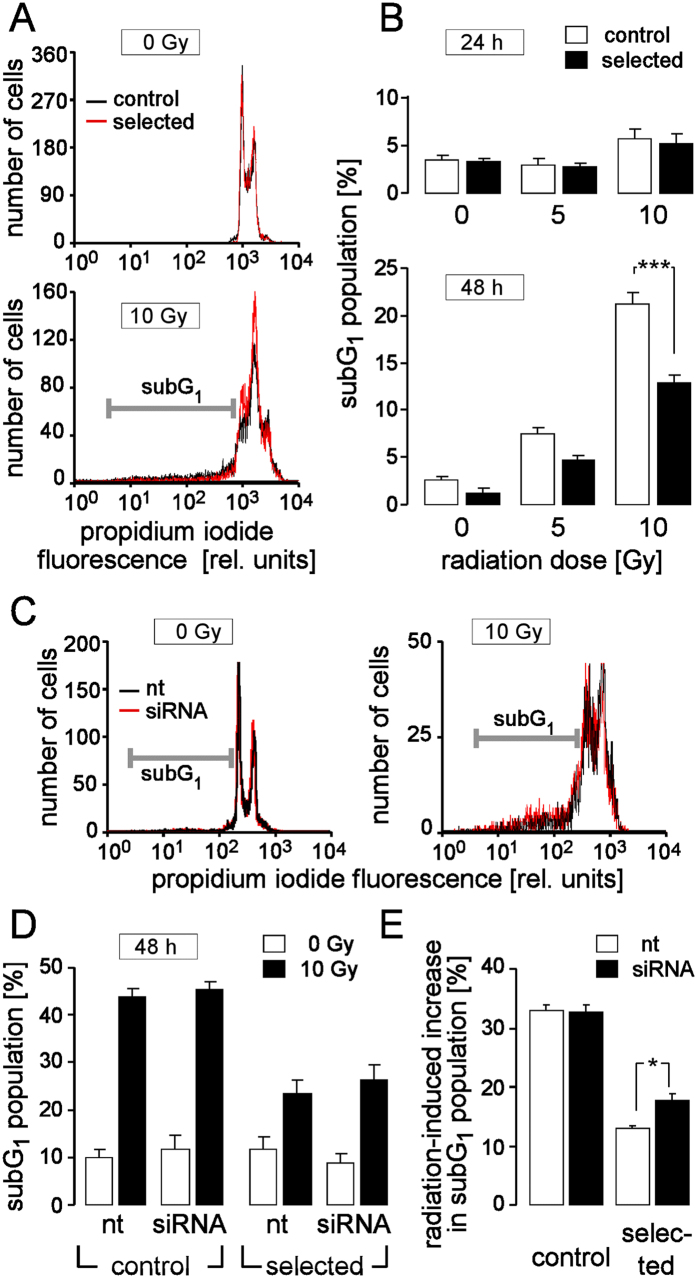
Cross resistance of H/R-adapted cultures against ionizing radiation. (**A**) Histograms showing the propidium iodide fluorescence of permeabilized control (black) and H/R-adapted (red) PT cells. Cells were recorded by flow cytometry 48 h after irradiation (under normoxic conditions) with 0 Gy (top) or 10 Gy (botom). The marker indicates the dead cells (sub G_1_ population). Percentage of dead cells (sub G_1_ population) in control (open bars) and H/R-adapted cultures (closed bars) 24 h (top) and 48 h (bottom) after irradiation (under normoxic conditions) with 0, 5, or 10 Gy. (**C**) Propidium iodide histograms of nt RNA- (black) and UCP-3 siRNA (red) transfected H/R-adapted PT cells 48 h after irradiation with 0 Gy (left) or 10 Gy (right). (**D**) Sub G_1_ population control (left) and H/R-adapted PT cultures (right) 96 h after transfection with nt or UCP-3 siRNA (as indicated) and 48 h after irradiation with 0 Gy (open bars) and 10 Gy (closed bars). Data are means ± SE, n = 9 in (**B**) or n = 6 in (**D**,**E**) from 3 (**B**) or 2 (**D**,**E**) cultures each determined in triplicates. * and *** indicate p ≤ 0.05 and p ≤ 0.001 (ANOVA), respectively.

Finally, we tested the possibility whether adaptation to H/R is accompanied by increased resistance against anti-cancer therapies such as radiation therapy. To this end, control and H/R-adapted cultures were irradiated under normoxia with a single dose of 0, 5, or 10 Gy, and subG_1_ population was recorded by flow cytometry (DNA staining of propidium iodide-permeabilized cells applying the Nicoletti protocol) as a measure of cell death 24 h and 48 h thereafter. As shown in Fig. 7, irradiated (10 Gy) H/R-adapted cells died significantly less than irradiated control cells as evident 48 h after irradiation. This might suggest that adaptation to H/R leads to partial cross-resistance against ionizing radiation possibly due to upregulation of DNA repair and oxidative defense (see Suppl. Fig. I). The latter might mitigate the radiation-caused cellular lesions.

To directly test for an involvement of UCP-3 in the acquired radioresitance of the H/R-adapted PT cultures, the effect of UCP-3 knockdown on radiogenic cell death was determined in control and H/R-adapted PT cultures. 48 h after irradiation (0 or 10 Gy) and 96 h after transfection with nt or UCP-3 siRNA, subG_1_ population of propidium iodide-stained cells was determined by flow cytometry (Fig. 7C,D). While having no effect on control cultures, UCP-3 knockdown increased slightly but significantly the fraction of dead cells caused by radiation (Fig. 7D,E).

## Discussion

UCP-3 uncoupling protein belongs to the mitochondrial anion transporter superfamily and is highly expressed in the mitochondrial inner membrane of brown adipose tissue, skeletal muscle and heart^6^,^7^. In the present study, selected, partially HR-resistant PT cultures differed from continuously normoxic grown control cultures by expression of genes involved in DNA repair, apoptosis and oxidative defense and by the ability to up-regulate mitochondrial UCP-3 uncoupling protein during H/R stress. Evidence for a functional significance of this UCP-3 upregulation for the partial H/R resistance in the selected cultures came from the observation that knockdown of UCP-3 by RNA interference significantly attenuated the H/R resistance in the selected cultures while having no effect in the control cultures.

An increasingly growing number of reports demonstrates that UCP-3 may lower mitochondrial ROS production by decreasing ΔΨ_m_ as a “mild uncoupler”. This mild uncoupling function of UCP-3 is in particular evident from the observation that UCP-3 knockout results in increased mitochondrial ROS formation and oxidative damage of isolated skeletal muscle mitochondria or permeabilized muscle cells^8^,^9^,^10^,^11^. Importantly, chemical uncoupling in UCP-3 knockout mitochondria partially mimics the function of UCP-3^11^. Moreover, UCP-3 knockout lowers fatty acid- or reactive aldehyde-stimulated increase in proton leak of the inner mitochondrial membrane^12^,^13^. Accordingly, overexpression of UCP-3 in myotubes decreases both ΔΨ_m_^14^,^15^,^16^ and mitochondrial ROS formation^15^.

In addition to these *in vitro* data, animal studies utilizing UCP-3 knockout or UCP-3 overexpressing mice demonstrated that UCP-3 decreases mitochondrial ROS-formation in fasted mice^10^ and protects from diet-induced obesity^17^ and insulin resistance^18^, two diseases associated with oxidative stress^15^. Moreover, downregulation of UCP-3 in rats that is associated with doxorubicin chemotherapy-induced heart failure improves efficiency of cardial ATP synthesis at an expense of increased mitochondrial ROS formation^19^. Finally, UCP-3 knockdown increased ischemia/reperfusion-induced mitochondrial ROS formation in the heart^20^. Again, chemical mitochondrial uncoupling partially compensated for UCP-3 function in the UCP-3 knock-out heart^20^. Combined these data provide overwhelming evidence that UCP-3 lowers stress-induced mitochondrial ROS formation by preventing excessive hyperpolarization of ΔΨ_m_.

The UCP-3-generated transports that may short-circuit the inner mitochondrial membrane, however, are still ill-defined. It has been suggested that UCP-3 may export pyruvate^21^ and fatty acids including lipid radicals^13^,^22^ from the mitochondrial matrix along their electrochemical gradients. Besides lowering ΔΨ_m_ and directly decreasing the concentration of lipid radicals in the matrix, pyruvate and fatty acids transports are thought to ensure an equilibrium between glycolysis and oxidative phosphorylation and to prevent Co-enzyme A shortage in the matrix and consecutive lipid-induced mitochondrial damage, respectively^23^,^24^. As matter of fact, UCP-3 upregulation increases the efficiency of fatty acid oxidation in exercising muscle^9^. Along those lines, in animal models and in human beings, fastening^25^,^26^, high fat diet^27^, or direct infusion of fatty acids^26^ have been demonstrated to upregulate UCP-3 expression which is consistent with a specific function of UCP-3 in switching the metabolism from glucose to fatty acid respiration. A recent meta-analysis demonstrates an association between the -55C/T polymorphism in the UCP-3 gene and obesity further suggesting such a UCP-3 function^28^.

Further triggers of UCP-3 expression include (anaerobic) exercise of skeletal muscle^9^,^29^ and ischemia/reperfusion of the heart^20^,^30^. Likewise, oxidative stress following inhibition of glutathione reductase have been demonstrated to upregulate UCP-3 expression in rat myocardium^31^ where UCP-3 has been demonstrated to be indispensable for ischemic preconditioning^20^. Moreover, reversible glutathionylation during oxidative stress is reportedly required to activate/inhibit UCP-3^32^. Together, these reported data indicate a function of oxidative-stress induced up-regulation of UCP-3 in mitigating ischemic/hypoxia-reperfusion/reoxygenation injury of heart and skeletal muscle.

In the present study, UCP-3 upregulation was paralleled by an attenuated H/R-mediated ΔΨ_m_ hyperpolarization and mitochondrial superoxide formation of the PT cultures hinting to a UCP-3 function similar to that proposed in ischemic heart or anaerobic muscle contraction. UCP-3 protein was also upregulated in the majority of specimens from human renal cell carcinoma (RCC) as compared to the protein abundance of co-resected normal kidney tissue. Since clear cell RCC originates from neoplastic transformation of proximal tubular cells it might be allowed to speculate that UCP-3 in RCC might also confer resistance to H/R. One might further speculate that UCP-3-mediated hypoxia tolerance and survival of the tumor cells in hypoxic areas indirectly confers resistance to chemo- and radiation therapy: the wash-in of chemotherapeutics is hampered by the malperfusion and the efficacy of ionizing radiation is lowered by the low oxygen pressure of hypoxic tumor areas. Along those lines, the TGCA query of the present study hint to the possibility that high UCP-3 expression by the RCC might be associated with poor prognosis.

Similar to the upregulation of UCP-3 by RCC in the present study, other UCPs such as UCP-1, UCP-2, UCP-4 or UCP-5 are reportedly upregulated in a number of aggressive human tumors (leukemia, breast-, colorectal-, ovarian-, bladder-, esophagus-, testicular-, kidney-, pancreatic-, lung-, and prostate cancer) where they are proposed to contribute to the malignant progression of tumors (for review see Ref. 4). Moreover, UCP-2 expression has been associated with paclitaxel resistance of p53 wildtype lung cancer, CPT-11 resistance of colon cancer, and gemcitabine resistance of pancreatic adenocarcinoma, non-small cell lung adenocarcinoma, or bladder carcinoma. Accordingly, experimental targeting of UCPs has been demonstrated to sensitize tumor cells to chemotherapy *in vitro*. (for review see Ref. 4).

In the present study, some RCC specimens exhibited elevated UCP-2 protein amounts as compared to normal renal tissue. UCP-1 protein, in sharp contrast, was downregulated in the RCCs. UCP-2 might exert a function in RCC similar to that proposed for UCP-3. This might be deduced from the TCGA database query of the present study which suggested that—likewise UCP-3—high RCC UCP-2 expression might be associated with bad prognosis.

In the present study, adaptation to H/R was accompanied by partial radioresistance. Knockdown of UCP-3 did, however, only slightly increase radiation-induced cell death of the H/R-adapted PT cultures. Upon UCP-3 knockdown the radiation-induced cell death of the control cultures was still much higher than in the selected cultures (see Fig. 7E) suggesting that radioresistance in the H/R-adapted PT cultures was substantially mediated by upregulation of DNA-repair and other oxidative defense mechanisms (see Suppl. Fig. I).

Ionizing radiation has been reported to upregulate UCP-2 expression in colon carcinoma cells^33^ and in a radiosensitive subclone of B cell lymphoma^34^, as well UCP-3 expression in rat retina^35^. Moreover, multi-resistant subclones of leukemia cells reportedly show higher UCP-2 protein expression, lower ∆Ψ_m_, lower radiation induced formation of reactive oxygen species and decreased DNA damage as compared to their parental sensitive cells^36^. Combined, these reported data hint to a possible function of UCPs in the development of radioresitance also in other tumor entities.

In conclusion, hypoxia/reoxygenation-tolerant PT cultures as well as renal cell carcinoma upregulate UCP-3 uncoupling protein in the inner mitochondrial membrane. UCP-3 upregulation attenuates at least in the *in vitro* PT model mitochondria-born oxidative stress during hypoxia/reoxygenation, and thus, confers partial hypoxia/reoxygenation resistance. Since tumor hypoxia promotes both, malignancy and therapy resistance, UCP-3 protein expression might be a prognostic and predictive marker in human neoplasms.

## Methods

### Selection of partially H/R-resistant proximal convoluted tubules (PT) cells

PT were microdissected from newborn mice as described elsewhere^37^. PT cells were immortalized by SV40 large T antigen transformation^38^ as accomplished by transfection with SV3 neo, selected in geneticin-containing medium and cultured on collagenated surfaces in equal quantities of DMEM and Ham’s F-12 (GIBCO) medium containing 29 mM NaHCO_3_ (GIBCO), 5% serum, 2.5 mM glutamine, 2.5 mg/l insulin, 2.5 mg/l transferrin, 15 nM sodium selenite (ITSS Supplement, Roche Diagnostics), 25 nM dexamethasone (Sigma), 5 ng/l epidermal growth factor (Calbiochem) and G418-BC (60U/ml, Biochrom). Four parallel cultures of PT cells were passaged (once per week) for 12 weeks. In this period of time, cells where weekly subjected to hypoxia (0.1% oxygen for 48 h starting 2–3 d after passaging the cells as applied by the BD GasPak EZ Pouch System,Becton and Dickinson). For control, further four PT cultures were grown under continuous normoxia, passaged twice weekly for 12 weeks. Thereafter, all cultures were passaged twice to increase cell number, aliquoted and frozen.

### H/R- and radiation-induced cell death

To test for an acquired hypoxia resistance, sub-confluent H/R-adapted and control cultures (from different selection cycles) were grown for 48 h under normoxia or hypoxia (0.1% oxygen) followed by 0.5, 24 or 48 h of reoxygenation. In further experiments, H/R-adapted and control cultures were irradiated with single doses of 0, 5, or 10 Gy photons by the use of a linear accelerator (LINAC SL25 Philips) at a dose rate of 4 Gy/min at room temperature under normoxia. Following irradiation, cells were post-incubated in supplemented medium for 24 h or 48 h. H/R-treated or irradiated cells were trypsinated and then permeabilized and stained (30 min at room temperature) with propidium iodide (PI) solution (containing 0.1% Na-citrate, 0.1% triton X-100, 10 μg/ml PI in phosphate-buffered saline, PBS), and the DNA amount was analyzed by flow cytometry (FACS Calibur, Becton Dickinson, Heidelberg, Germany, 488 nm excitation wavelength) in fluorescence channel FL-2 (logarithmic scale, 564–606 nm emission wavelength). Dead cells with degraded DNA were defined by the sub G_1_ population of the PI histogram. Data were analyzed with the FCS Express 3 software (De Novo Software, Los Angeles, CA, USA).

### Inner mitochondrial membrane potential (ΔΨ_m_)

To determine ΔΨ_m_, hypoxia (48 h)/reoxygenation (24 h)-subjected as well as normoxic grown H/R-adapted and control PT cultures were trypsinated, washed and incubated for 30 min at room temperature in a NaCl solution (in mM: 125 NaCl, 5 d-glucose, 5 KCl, 1 MgCl_2_, 1 CaCl_2_, 32 N-2-hydroxyethylpiperazine-N-2-ethanesulfonic acid (HEPES) titrated with NaOH to pH 7.4) containing the ΔΨ_m_ specific dye tetramethylrhodamine ethyl ester perchlorate (TMRE, 25 nM, Invitrogen). ΔΨ_m_ was analyzed by flow cytometry in FL-2 in the absence or presence of the proton ionophore carbonyl cyanide-3-chlorophenylhydrazone (CCCP, 1 μM).

### Formation of reactive oxygen species (ROS)

To test for mitochondrial production of superoxide anion, control or H/R-adapted PT cells were pretreated (normoxia or hypoxia (48 h)/reoxygenation (24 h)), detached, incubated for 10 min at 37 °C in NaCl solution (see above) containing 5 μM of the superoxide anion-sensitive dye MitoSOX, Invitrogen), and ROS-specific fluorescence was recorded by flow cytometry in Fl-2. To test for fluorescence dye loading, control samples were oxidized (1 mM *tert*-butylhydroperoxide) for 30 min and recorded.

### Quantitative RT-PCR

Messenger RNAs of H/R-adapted and control PT cultures (normoxic and after hypoxia (48 h)/reoxygenation (24 h)) were isolated (Qiagen RNA extraction kit, Hilden, Germany) and reversely transcribed in cDNA (RT^2^ First Strand Kit, SABiosciences, Qiagen). mRNA fragments were amplified by the use of an “oxidative stress” and an “apoptosis” PCR array (RT Profiler PCR Array, SABiosciences, Qiagen, Hilden, Germany) and a Roche Light Cycler 480. C_*t*_ (threshold cycle) values of the PCR amplifications were normalized to that of the housekeeper genes. Mouse UCP-3 and GAPDH specific cDNAs were amplified by QuantiTect Primer Assays (#QT00115339 and QT01658692, respectively, Qiagen).

### Transfection with siRNA

Adherent PT cells were grown in normal medium and transfected at 60% confluence with a transfection reagent (TransIT-TKO, Mirus Bio, Madison, WI, USA) according to the manufacturer’s instructions. UCP-3 siRNA and non-targeting siRNA (ON-TARGETplus SMARTpool, ON-TARGET Non-targeting siRNA, ThermoScientific Dharmacon, Chicago, IL, USA) were used at a final concentration of 50 nM. Transfection efficiency and viability was determined by transfecting the cells with 400 nM green fluorescence siGLO siRNA (ThermoScientific Dharmacon) followed by propidium iodide exclusion dye and flow cytometric analysis (data not shown). Immediately after transfection, cells were cultured either under normal conditions or hypoxic (48 h)/reoxygenation (24 h). Downregulation of UCP-3 was controlled by immunoblotting.

### Immunoblotting

Following normoxia or hypoxia (48 h)/reoxygenation (24 h) H/R-adapted and control PT cultures as well as frehly frozen specimens from dissected human renal cell carcinoma with adjacent normal renal tissue. Patients gave informed consent and the experiments were approved by the ethical committee of the Faculty of Medicine, University of Tübingen (Ethik-Kommission der Medizinischen Fakultät und am Universitätsklinikum Tübingen, Gartenstrasse 47, 72074 Tübingen, Germany) and carried out in accordance with the approved guidelines. Specimens were lysed in a buffer (containing in mM: 50 HEPES pH 7.5, 150 NaCl, 1 EDTA, 10 sodium pyrophosphate, 10 NaF, 2 Na_3_VO_4_, 1 phenylmethylsulfonylfluorid (PMSF) additionally containing 1% triton X-100, 5 μg/ml aprotinin, 5 μg/ml leupeptin, and 3 μg/ml pepstatin) and separated by SDS-PAGE under reducing condition. Segregated proteins were electro-transferred onto PVDF membranes (Roth, Karlsruhe, Germany). Blots were blocked in TBS buffer containing 0.05% Tween 20 and 5% non-fat dry milk for 1 h at room temperature. The membrane was incubated overnight at 4 °C with rabbit anti-UCP3 antibody (#ab3477, Abcam, Cambridge, UK). Equal gel loading was verified by an antibody against β-actin (mouse anti-β-actin antibody, clone AC-74, Sigma #A2228 1:20,000) or GAPDH (mouse anti-GAPDH, #ab8245 clone 6C5, Abcam, 1:20,000). Antibody binding was detected with a horseradish peroxidase-linked goat anti-rabbit or horse anti-mouse IgG antibody (Cell Signaling #7074 and #7076, New England Biolabs GmbH, Frankfurt am Main, Germany, 1:1000 and 1:2000 dilution in TBS-Tween/5% milk, respectively) incubated for 1 h at room temperature and enhanced chemoluminescence (ECL Western blotting analysis system, GE Healthcare/Amersham-Biosciences, Freiburg, Germany). Where indicated protein levels were quantified by densitometry using ImageJ software (ImageJ 1.40 g NIH, USA). UCP-3 expression of the tumor samples were categorized using an arbitrary score from 0 (not expressed, see Fig. 6B, 1^st^ lane) to 4 (highest expression, see Fig. 6B, 8^th^ lane).

### Statistics

Given data are means ± standard error (SE). Differences between experimental groups were assessed by (Welch-corrected) two-tailed student t-test or ANOVA where appropriate. P values of ≤0.05 were defined significant.

## Additional Information

**How to cite this article**: Braun, N. *et al.* UCP-3 uncoupling protein confers hypoxia resistance to renal epithelial cells and is upregulated in renal cell carcinoma. *Sci. Rep.* **5**, 13450; doi: 10.1038/srep13450 (2015).

## Supplementary Material

Supplementary Information
