# Supplementary material for: UCP-3 uncoupling protein confers hypoxia resistance to renal epithelial cells and is upregulated in renal cell carcinoma
Source: Sci Rep. 2015 Aug 25;5:13450. doi: 10.1038/srep13450 (PMC4548255; doi:10.1038/srep13450)
Supplement: Supplementary Information [file srep13450-s1.pdf]

Supplementary Material

UCP-3 uncoupling protein confers hypoxia resistance to renal epithelial cells and is upregulated in renal cell carcinoma

Norbert Braun, Dominik Klumpp, Jörg Hennenlotter, Jens Bedke, Christophe Duranton, Martin Bleif, and Stephan M. Huber

H/R adaptation seemed to be associated with upregulation of mRNAs involved in oxidative defense, DNA-repair, apoptosis, and mitochondrial uncoupling. The comparison of mRNA abundances (quantitative RT-PCR microarrays) in control and H/R-adapted PT cultures after normoxic culture conditions and upon hypoxia (48 h)/ reoxygenation (24 h) by quantitative RT-PCR microarrays suggested that H/R-adapted PT cultures constitutively upregulated the anti-apoptotic survivin, the caspase-recruitment domain-containing proteins PYCARD and Bcl-10, the antioxidative-defense associated thioredoxin reductase and superoxide dismutases 2 and -3, the DNA repair enzyme Ataxia telangiectasia and rad3 related (ATR), as well as the actin filament-stabilizing tropomodulin 1. In addition, the H/R adaptation induced the constitutive downregulation of the presumably antiapoptotic cytoglobin (Suppl. Fig. I, open bars).

Moreover, acute H/R induced higher mRNA abundances in the H/R-adapted as compared to the control PT cultures of the pro-apoptotic FADD (Fas-associated via death domain) and caspase-6, the stress-inducible Trp53inp1 (tumor protein p53-inducible nuclear protein 1), and the uncoupling protein UCP-3. Glutathione peroxidase 2, in contrast, was more induced during acute H/R in control than in H/R-adapted PT cultures (Suppl. Fig. I, closed bars).

High UCP-2 and UCP-3 mRNA abundance in clear cell RCC might be associated with bad prognosis. To test whether other uncoupling proteins are upregulated by RCCs like UCP-3, UCP-1 and 2 protein abundances were determined in the resection material of 4 human clear cell RCCs and adjacent normal renal tissue and compared to that of UCP-3. UCP-1 protein could be detected in normal renal tissue but was absent in RCC. In contrast, UCP-2 and UCP-3 proteins were low in normal renal tissue but up-regulated in 2 (UCP-2) and 4 (UCP-3) out of 4 tumors (Suppl. Fig. IIA).

To get a hint, whether UCPs expression might be associated with RCC malignancy, the ccRCC database of TCGA (RNA Seq V2 RSEM data [1]) was queried for UCP-1, -2, and -3 mRNA abundance and patient survival via the cBIOportal Web resource [2,3]. Applying a cut-off of +1.28 Z-scores revealed 43, and 45 out of 532 tumors with elevated UCP-3 and UCP-2 mRNA abundance, respectively (the Z score of the UCP mRNA abundance in an individual ccRCC is calculated by the number of standard deviations the individual mRNA abundance differs from the mean value of all ccRCC that are diploid for the UCP gene). For UCP-1, a cut-off of 1.0 Z score yielded 49 ccRCCs with elevated UVP-1 mRNA.

As a result, high abundance of UCP-2 in the ccRCC significantly ($P \leq 0.001$, Fisher exact test) co-occurred with high abundance of UCP-3. Moreover, overall survival of RCC patients with high UCP-3 (Supplementary Fig. IIB, red) or high UCP-2 mRNA abundance in the tumor (Suppl. Fig. IIC, red) was shorter than that of RCC patients with “middle-rate” (i.e., mRNA abundance < mean + 1.28 Z scores) UCP-2 or UCP-3 mRNA abundance (Suppl. Fig. IIB, C, blue). In accordance to the protein data of the RCC resection material (Suppl. Fig. IIA), mRNA data of the TCGA ccRCC database did not suggest co-occurrence of high UCP-1 mRNA abundance neither with high UCP-2 nor with high UCP-3 mRNA abundance. In

addition, high UCP-1 mRNA abundance of the tumor was not associated with altered overall survival of ccRCC patients ($p = 0.89$, data not shown). Together, these clinical data might hint to the functional redundancy of UCP-2 and -3 in ccRCC, and a prognostic value of UCP-2 and/or UCP-3 abundance for overall survival of ccRCC patients.

References

1 Comprehensive molecular characterization of clear cell renal cell carcinoma. *Nature* **499**, 43-49 (2013).

2 Gao, J. *et al.* Integrative analysis of complex cancer genomics and clinical profiles using the cBioPortal. *Sci Signal* **6**, pl1 (2013).

3 Cerami, E. *et al.* The cBio cancer genomics portal: an open platform for exploring multidimensional cancer genomics data. *Cancer Discov* **2**, 401-404 (2012).

Figures and Figure Legends

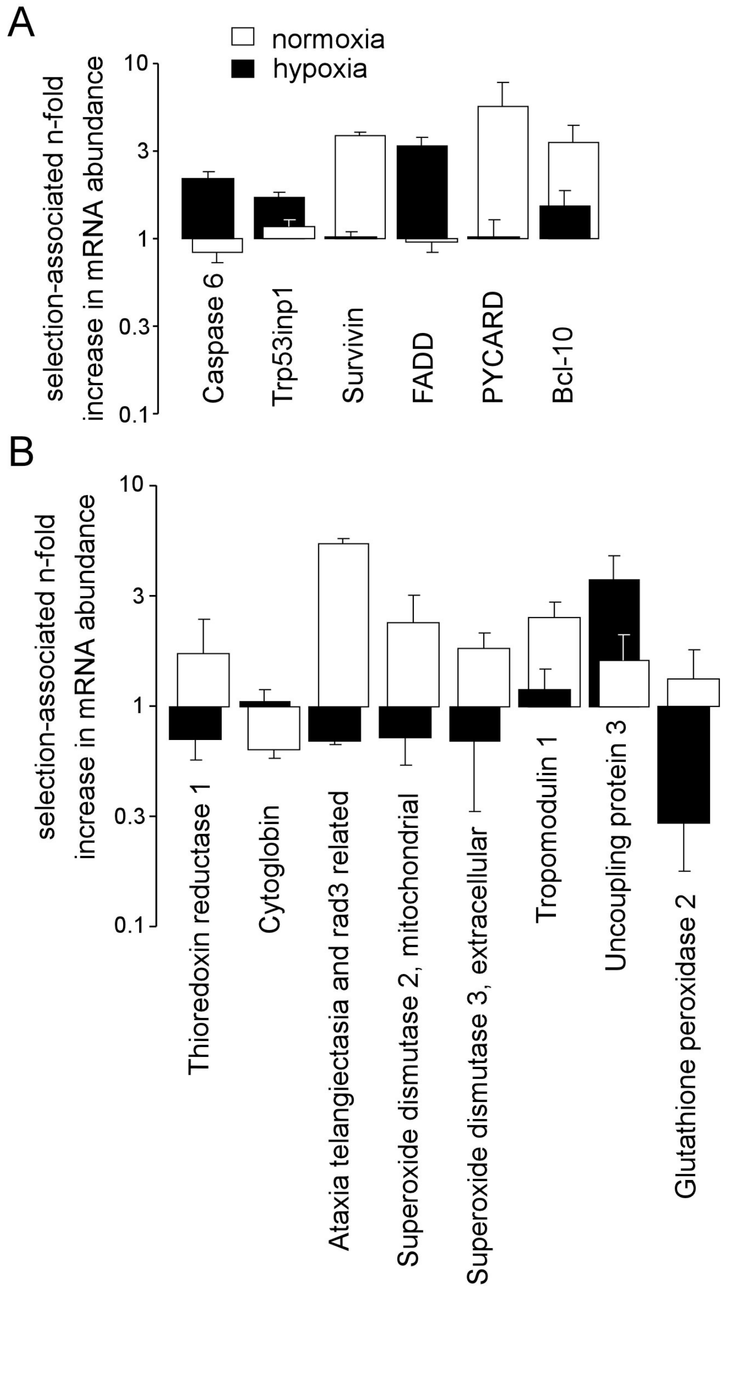

Suppl. Fig. I. Mean (± SE) mRNA abundances of three H/R-adapted cultures under normoxia (open bars) or after hypoxia (48 h)/ reoxygenation (24 h; closed bars) normalized to the pooled mRNA abundance of the respective control cultures as determined by quantitative RT-PCR array on “apoptosis” (A) and “oxidative stress” (B) genes.

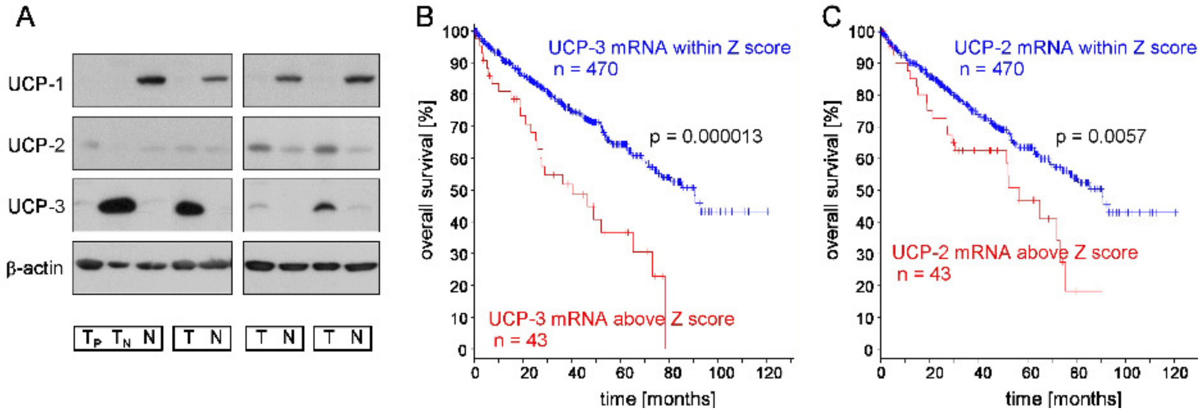

Supp. Fig. II. Possible prognostic value of UCP-3 or -2 mRNA abundance for the survival of ccRCC patients. A. Immunoblot of protein lysates from human RCC and adjacent normal tissue probed against UCP-1 (#ab57687, Abcam), UCP-2 (#ab67241, Abcam), UCP-3 (#ab3477, Abcam), and for loading control against β-actin (N: normal renal tissue, T: tumor without defined origin. T_N: tumor close to necrotic area, T_P: tumor at the periphery. Boxes indicate samples which originated from the same kidney). **B, C.** Kaplan Meier plots showing the overall survival of ccRCC patients with high UCP-3 (B, red) or high UCP-2 mRNA-expressing tumors (C, red) in comparison with the respective control cohorts (blue). Patient numbers and p-values (log rank test) are indicated.
